# Hyperreflective choroidal foci may predict pachychoroid macular atrophy development in central serous chorioretinopathy

**DOI:** 10.1038/s41433-026-04277-8

**Published:** 2026-02-05

**Authors:** Maria Grazia Pignataro, Alba Chiara Termite, Enrico Borrelli, Giacomo Boscia, Michele Reibaldi, Luisa Micelli Ferrari, Giulia Ribezzi, Alice Carra, Stefano Dore, Federica Evangelista, Giovanni Alessio, Francesco Boscia, Pasquale Viggiano

**Affiliations:** 1 Department of Translational Biomedicine Neuroscience, University of Bari “Aldo Moro”, Bari, Italy; 2 Department of Ophthalmology, “City of Health and Science” Hospital, Turin, Italy; 3 Department of Medicine, Surgery and Pharmacy, University of Sassari, Sassari, Italy; 4 Department of Ophthalmology, “Di Venere” Hospital, Bari, Italy

**Keywords:** Microbiology, Predictive markers

## Abstract

**Purpose:**

To evaluate hyperreflective choroidal foci (HCF) in Sattler’s and Haller’s layers as predictive biomarkers for treatment response and pachychoroid macular atrophy (pMA) development in chronic central serous chorioretinopathy (CSC).

**Methods:**

Retrospective analysis of 70 treatment-naïve patients with recurrent CSC classified according to Chhablani’s criteria. HCF were quantified separately in choroidal layers using spectral-domain OCT at baseline and 12-month follow-up. Patients received photodynamic therapy (*n* = 20), eplerenone (*n* = 16), or subthreshold micropulse laser (*n* = 34). Primary outcomes included treatment response (complete fluid resolution) and pMA development.

**Results:**

At baseline, no significant differences in HCF counts existed between future responders (*n* = 36) and non-responders (*n* = 34). At 12 months, responders showed significant HCF reduction in Sattler’s layer (−9.17 foci, *p* = 0.001) and Haller’s layer (−3.19 foci, *p* = 0.039), while non-responders demonstrated increased Sattler’s foci (+4.62, *p* = 0.041). pMA developed in 15 patients (21.4%), more frequently in non-responders (32.4% vs 11.1%, *p* = 0.001). Baseline total HCF count was the strongest predictor of pMA development (β = 0.465, *R*² = 0.324, *p* < 0.001), with final HCF counts showing even stronger associations (β = 0.512, *R*² = 0.348, *p* < 0.001).

**Conclusions:**

Layer-specific HCF quantification provides valuable prognostic information for treatment response and pMA risk in chronic CSC. These biomarkers may guide therapeutic decisions and identify patients requiring closer monitoring for atrophy development.

## Introduction

Central serous chorioretinopathy (CSC) is a multifactorial disorder characterized by serous detachment of the neurosensory retina, often associated with retinal pigment epithelium (RPE) dysfunction and choroidal vascular abnormalities [1, 2]. While acute CSC frequently resolves spontaneously, chronic forms present significant therapeutic challenges and carry substantial risk for permanent visual impairment due to progressive retinal changes, including the development of retinal atrophy [3–5].

Recent advances in optical coherence tomography (OCT) imaging have enhanced our understanding of CSC pathophysiology, revealing complex interactions between choroidal vasculature, RPE, and neurosensory retina [2, 6, 7]. Among the various morphological features observed in CSC, hyperreflective choroidal foci (HCF) have emerged as potentially significant biomarkers [8]. These discrete, hyperreflective structures within the choroidal stroma have been associated with inflammatory processes, vascular dysfunction, and disease activity in various chorioretinal conditions [9, 10].

The choroidal architecture consists of distinct vascular layers, with Sattler’s layer containing medium-sized vessels and Haller’s layer comprising larger choroidal vessels. Recent studies suggest that pathological changes in these layers may contribute differentially to CSC pathogenesis and progression [6]. However, the specific role of hyperreflective foci in each choroidal layer and their relationship with treatment outcomes remains incompletely understood.

Of particular clinical importance is the development of retinal atrophy in chronic CSC, which has been increasingly recognized as a cause of irreversible visual loss [11, 12]. Recent studies have described this entity as pachychoroid macular atrophy (pMA), characterized by complete RPE and outer retinal atrophy in the setting of pachychoroid phenotype [13]. Understanding predictive factors for atrophy development is crucial for optimizing treatment strategies and patient counseling, as the progression rate and characteristics of pMA in CSC appear distinct from those of age-related macular degeneration-related geographic atrophy.

Current treatment options for chronic CSC include photodynamic therapy (PDT) and laser treatment in cases with extrafoveal leakage. Additionally, subthreshold micropulse laser therapy (SMLT) and eplerenone (EPL) have been proposed by some authors as alternative approaches, each targeting distinct mechanisms involved in the disease pathophysiology [14]. However, predicting individual treatment response and identifying patients at high risk for atrophy development remains challenging. Reliable biomarkers that can guide therapeutic decisions and provide prognostic information are urgently needed.

This study aims to investigate the prognostic value of layer-specific hyperreflective choroidal foci quantification in chronic CSC. We hypothesize that the number and distribution of HCF in Sattler’s and Haller’s layers can predict treatment response and identify patients at increased risk for developing retinal atrophy.

## Methods

### Study participants

This retrospective observational study was conducted at the Medical Retina Clinic of the Department of Translational Biomedicine and Neuroscience at the Aldo Moro University of Bari, Italy. We analyzed 70 treatment-naïve patients diagnosed with chronic CSC between January 2022 and December 2024. The study was conducted in accordance with the tenets of the Declaration of Helsinki. As per Italian regulations for retrospective studies, formal approval from an ethics committee was not required, but the institutional ethics committee was informed about the study.

Inclusion criteria were: age ≥18 years, diagnosis of CSC confirmed by multimodal imaging including fluorescein angiography (FA), indocyanine green angiography (ICGA), and OCT classification as recurrent CSC according to Chhablani’s criteria [3] characterized by current subretinal fluid presence with documented history or imaging evidence of previously resolved episodes, and when both eyes met the inclusion criteria, the right eye was selected for analysis. According to Chhablani’s multimodal imaging-based classification system [3], all included eyes were classified as simple or complex CSC based on the extent of RPE alteration (threshold of 2-disc area diameter).

Exclusion criteria included: presence of cataract affecting image quality, previous vitrectomy or any other type of intraocular surgery, history of any treatment for CSC including laser therapy, photodynamic therapy, anti-VEGF injections, or systemic medications such as eplerenone, current or systemic corticosteroid use during the follow-up year, concurrent maculopathies including age-related macular degeneration (AMD) or diabetic retinopathy (DR) [15–17], pathologic myopia defined as axial length greater than 26.5 mm or spherical equivalent exceeding −6.0 diopters, macular neovascularization resulting from other causes, pregnancy or nursing status, significant media opacities preventing high-quality imaging acquisition [18, 19], and inability to provide informed consent or complete follow-up examinations.

Treatment response was defined according to Chhablani’s classification [3] as resolved CSCR, characterized by the complete absence of SRF on structural OCT at 12-month follow-up examination. Patients were categorized as responders (resolved CSC) or non-responders (persistent or recurrent CSC with ongoing SRF) based on this classification system.

Of the initial 87 patients screened, 17 were excluded: 12 patients (13.8%) were excluded because the choroid was not fully visible in all B-scans, and 5 patients (5.7%) were excluded for other reasons, including poor image quality (*n* = 3) and incomplete follow-up data (*n* = 2).

### Disease duration assessment

Disease duration was meticulously calculated through a comprehensive review of medical records, previous imaging studies, and detailed patient history. The onset was determined from the first documented evidence of serous retinal detachment on OCT imaging or the earliest documented symptoms consistent with CSC, including metamorphopsia, central scotoma, or micropsia, corroborated by clinical findings. For patients presenting with recurrent episodes, disease duration encompassed the entire disease history, including periods of resolution and recurrence.

### Examinations

All participants underwent a comprehensive ophthalmological evaluation, including best-corrected visual acuity measurement using Early Treatment Diabetic Retinopathy Study charts converted to logMAR units for statistical analysis, slit-lamp biomicroscopy with detailed anterior segment examination, Goldman applanation tonometry for intraocular pressure measurement, and dilated fundus examination using indirect ophthalmoscopy and fundus contact lens examination.

Multimodal imaging was performed using FA, ICGA, fundus autofluorescence (FAF), structural OCT, and OCT Angiography (OCTA). FA and ICGA were conducted using the Heidelberg Spectralis HRA + OCT system (Heidelberg Engineering, Heidelberg, Germany) to confirm CSC diagnosis, evaluate choroidal vascular hyperpermeability patterns, assess RPE dysfunction, and exclude concurrent macular neovascularization.

### Image acquisition and analysis

All structural OCT imaging was performed using the Heidelberg Spectralis HRA + OCT system (Heidelberg Engineering, Heidelberg, Germany) with the following standardized acquisition protocol: spectral-domain OCT imaging session included 19 horizontal B-scans covering approximately a 5.5 × 4.5 mm area centered on the fovea, with each B-scan composed of 25 averaged OCT images to optimize image quality and reduce noise artifacts [20]. A minimum signal strength of 25 was required for OCT images to be included in the analysis, as recommended by the manufacturer. Only volumetric scans in which the choroid was fully visible in all B-scans were included for analysis to ensure accurate choroidal layer identification and foci quantification.

### Hyperreflective choroidal foci quantification

**Fig. 1:**
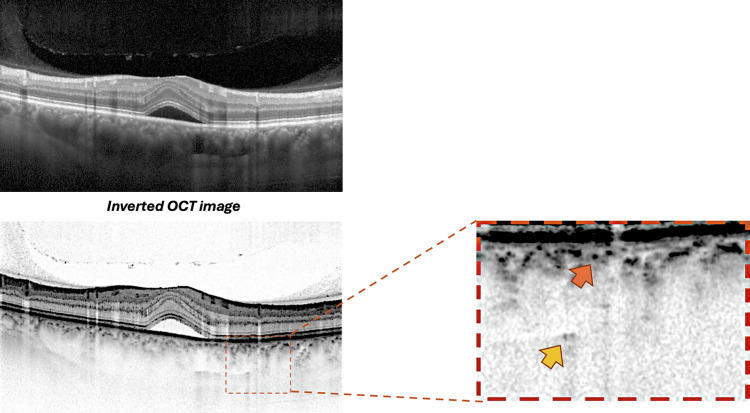
Methodology for hyperreflective choroidal foci quantification. Representative OCT B-scan demonstrating the standardized protocol for hyperreflective choroidal foci identification and quantification. Top panel: Standard OCT B-scan of a patient with CSC showing subretinal fluid and choroidal thickening. Middle panel: Inverted OCT image, enhancing visualization of choroidal hyperreflective foci through improved contrast. Right panel: High-magnification view of the choroidal area showing hyperreflective foci in Sattler’s layer (orange arrow) and Haller’s layer (yellow arrow).

Prior to quantifying HCF, two experienced graders (PV and EB) reached consensus on identifying and delineating each choroidal layer in the OCT sections following established methodology [9]. Haller’s layer was defined as the outermost choroidal layer housing the large choroidal vessels, while Sattler’s layer was described as the region containing medium-sized choroidal vessels, appearing as medium-sized, less intense spaces surrounded by more intense stroma (Fig. 1). The choriocapillaris was characterized as the less reflective layer situated above Sattler’s layer and encompassing Bruch’s membrane as well [6, 21].

HCFs were defined as round or oval areas with high reflectivity, appearing black or white in color on white-over-black and black-over-white images, respectively, with a diameter between 10 and 50 micrometers [9, 10, 20]. Two independent observers (PV and EB) counted the number of HCF in different layers of the choroid, including the choriocapillaris-Sattler’s layer complex, and Haller’s layer (Fig. 1). The identification and counting were performed on high-magnification white-over-black spectral-domain OCT images to improve the identification of hyperreflective foci in the choroid [9, 10]. For hyperreflective foci quantification, both readers counted foci in each designated choroidal layer following the standardized protocol [9, 10]. The values provided by both graders were utilized to evaluate intergrader repeatability. The average of the ratings from the two graders was incorporated into the final analysis. One of the graders (PV) conducted the grading process twice on different days to determine intragrader repeatability. In cases where foci counts differed by more than 20%, a third experienced reader (FB) performed the quantification, and the median value was used for analysis. The intraclass correlation coefficient was calculated to assess inter-observer agreement for foci quantification.

### Treatment protocols

Three distinct treatment modalities were employed in this study.

1. PDT was performed using a half-dose verteporfin protocol with intravenous administration of 3 mg/m² verteporfin (Visudyne, Novartis Pharma AG, Basel, Switzerland) followed by laser activation at 689 nm wavelength with reduced fluence of 25 J/cm² over 83 s, targeting areas of choroidal hyperpermeability identified on ICGA [22, 23].
2. Oral eplerenone therapy consisted of continuous daily administration of 50 mg eplerenone (Inspra, Pfizer Inc., New York, NY, USA) with mandatory serum potassium level monitoring performed bimonthly for the first 6 months and subsequently every 3 months throughout the treatment period to prevent hyperkalemia [24, 25].
3. SMLT (Navilas® 577 s system) was delivered using standardized parameters, including power setting of 200 mW, pulse duration of 200 ms, and duty cycle of 5%, applied directly to areas of foveal subretinal fluid identified on optical coherence tomography, with treatment spots covering the entire area of subretinal fluid accumulation in the foveal region [26, 27].

Treatment selection in this retrospective cohort was based on clinical assessment and established practice guidelines. PDT was generally selected for patients with diffuse choroidal hyperpermeability on ICGA and prominent pachychoroid features. SMLT was preferred for cases with focal or extrafoveal leakage on FA and in patients preferring non-invasive approaches. Eplerenone was offered to patients declining invasive procedures, those with contraindications to PDT, or as initial therapy in less severe presentations. Final treatment decisions incorporated patient preferences, contraindications, and cost considerations.

### Follow-up protocol and outcome assessment

**Fig. 2:**
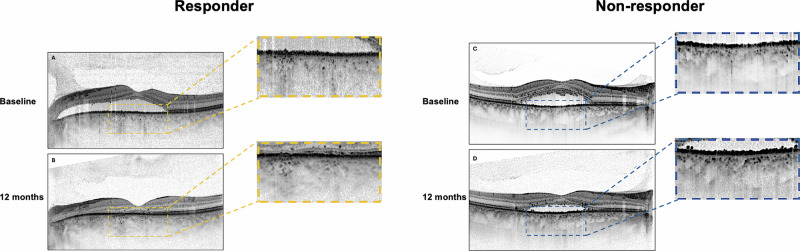
Differential evolution of hyperreflective choroidal foci in treatment responders versus non-responders. Representative cases demonstrating contrasting patterns of hyperreflective choroidal foci evolution during 12-month follow-up. Left panels: Treatment responder showing baseline OCT scan (**A**) with multiple hyperreflective foci and 12-month follow-up (**B**) demonstrating significant foci reduction with complete subretinal fluid resolution. Right panels: Treatment non-responder displaying baseline OCT (**C**) and 12-month follow-up (**D**) with increased foci density and persistent subretinal fluid. Magnified views: High-resolution details of choroidal foci distribution at both timepoints, illustrating the divergent temporal evolution patterns. Responders demonstrated a mean foci reduction of 9.17 in Sattler’s layer, while non-responders showed a mean increase of 4.62 foci.

All patients were retrospectively analyzed for follow-up data at 12 months after treatment initiation. Medical records were reviewed for comprehensive ophthalmological examinations, including visual acuity assessment, fundus examination, and OCT imaging. Treatment response was rigorously evaluated at 12-month follow-up and defined as the complete absence of subretinal and intraretinal fluid on structural OCT examination. Non-response is defined as a reduction but not complete resolution of fluid or stable or increased fluid accumulation (Fig. 2).

Primary and secondary outcome measures were systematically evaluated using structural OCT at baseline and 12-month follow-up.

#### Quantitative parameters

- *Central retinal thickness (CRT)* was automatically measured by the device software as the distance between the internal limiting membrane and the retinal pigment epithelium at the foveal center [27].
- *Subfoveal choroidal thickness (SFCT)* was manually measured from the outer border of the RPE to the choroidal-scleral interface directly beneath the foveal center using the built-in calliper tool [24].
- Subretinal fluid (SRF*) height* was measured as the maximum vertical distance between the neurosensory retina and RPE [25].

#### Qualitative parameters

**Fig. 3:**
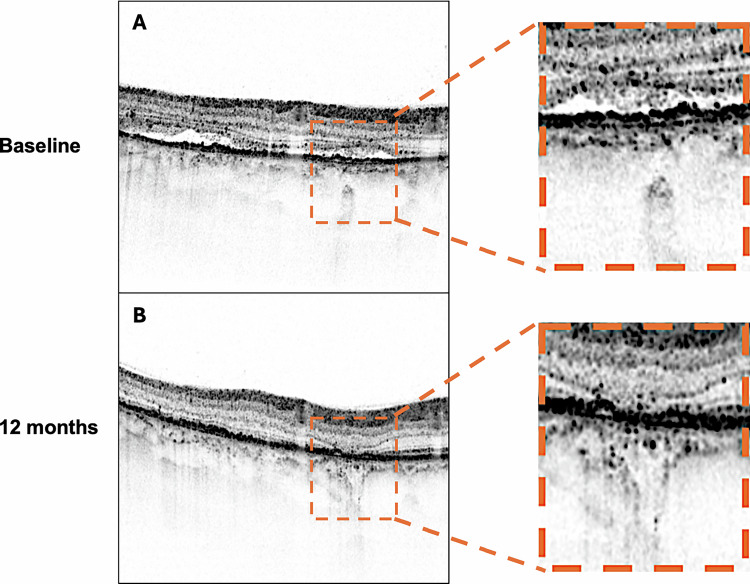
Development of pachychoroid macular atrophy in a patient with high baseline hyperreflective choroidal foci. Longitudinal case demonstrating the predictive value of baseline hyperreflective choroidal foci for pachychoroid macular atrophy development. **A** Baseline inverted OCT image showing numerous hyperreflective choroidal foci throughout Sattler’s and Haller’s layers, with preserved outer retinal architecture and RPE integrity. **B** 12-month follow-up revealing development of pachychoroid macular atrophy characterized by complete loss of outer retinal layers and retinal pigment epithelium, appearing as areas of increased choroidal transmission. Magnified views: Detailed visualization of baseline foci density and subsequent atrophic changes.

- *Subretinal hyperreflective material (SHRM)* was defined as hyperreflective deposits located between the neurosensory retina and retinal pigment epithelium, distinct from subretinal fluid [28].
- *Pachychoroid macular atrophy (pMA)* was systematically assessed using both structural OCT and FAF imaging. pMA was defined as a complete loss of the outer retinal layers, including photoreceptors and RPE, appearing as areas of increased transmission into the choroidal layers on OCT and corresponding to well-demarcated hypoautofluorescent regions on FAF imaging that developed during the follow-up period in eyes without baseline atrophy (Fig. 3) [11, 13].

### Statistical analysis

Statistical analysis was conducted using SPSS Statistics version 28.0 (IBM Corp., Armonk, NY, USA). The Shapiro–Wilk test was employed to assess the normality of continuous variables. Descriptive statistics were presented as mean ± standard deviation for normally distributed continuous variables and as frequencies with percentages for categorical variables. Between-group comparisons for continuous variables were performed using an independent samples t-test for normally distributed data and a Mann–Whitney U test for non-normally distributed data. Within-group comparisons for paired measurements were conducted using a paired *t*-test for normally distributed differences and a Wilcoxon signed-rank test for non-normally distributed differences. Categorical variables were compared using the χ^2^ test or Fisher’s exact test when expected cell counts were less than five. Linear regression analysis was performed to identify predictors of atrophy development, with results reported as standardized beta coefficients, R-squared values, and corresponding p-values. Statistical significance was established at *p* < 0.05, and all tests were two-tailed.

## Results

### Demographic and baseline characteristics

A total of 70 patients were included in this retrospective analysis. The study population comprised 42 males (60.0%) and 28 females (40.0%) with a mean age of 53.4 ± 12.3 years (range: 28–74 years). According to Chhablani’s classification, 46 patients (65.7%) were classified as simple CSC and 24 patients (34.3%) as complex CSCR [3]. All patients presented with recurrent CSC characterized by current SRF with documented history of previous episodes, with 38 patients (54.3%) demonstrating persistent features defined as SRF duration exceeding 6 months (Table 1).

**Table 1:** Baseline demographic and clinical characteristics.

Characteristic	Value
Number of patients	70
Mean age (years) ± SD	53.4 ± 12.3
Male gender, *n* (%)	42 (60.0)
Simple CSC, *n* (%)	46 (65.7)
Complex CSC, *n* (%)	24 (34.3)
Persistent features, *n* (%)	38 (54.3)
Baseline BCVA (decimal) ± SD	0.52 ± 0.14
Sattler’s layer foci (*n*) ± SD	56.45 ± 26.43
Haller’s layer foci (*n*) ± SD	7.69 ± 9.58
Central retinal thickness (μm) ± SD	412.4 ± 131.2
Choroidal thickness (μm) ± SD	420.4 ± 100.4
Subretinal fluid height (μm) ± SD	301.6 ± 152.8
SHRM present, *n* (%)	37 (52.8)

Data are presented as mean ± standard deviation for continuous variables and as number (percentage) for categorical variables.

*CSC* central serous chorioretinopathy, *BCVA* best-corrected visual acuity, *SHRM* subretinal hyperreflective material, *SD* standard deviation.

Baseline best-corrected visual acuity was 0.52 ± 0.14 decimal (approximately 20/40 Snellen equivalent). Mean CRT measured 412.4 ± 131.2 μm, while SFCT was 420.4 ± 100.4 μm. SRF height averaged 301.6 ± 152.8 μm. At baseline examination, SHRM was present in 37 patients (52.8%). No patients demonstrated pMA at baseline evaluation.

### Treatment distribution and response analysis

In our retrospective cohort, patients had been treated with three different modalities: 20 patients (28.6%) had received PDT, 16 patients (22.9%) had received eplerenone therapy, and 34 patients (48.6%) had undergone SMLT. At the 12-month follow-up evaluation, 36 patients (51.4%) were retrospectively classified as responders according to Chhablani’s criteria, having achieved complete resolution of SRF. The remaining 34 patients (48.6%) were classified as non-responders, showing persistent or recurrent SRF at the final evaluation (Table 2).

**Table 2:** Treatment outcomes by response group.

	Responders (*n* = 36)	Non-Responders (*n* = 34)	*P*-value
**Treatment distribution**			
PDT, *n* (%)	9 (25.0)	11 (32.4)	0.742
Eplerenone, *n* (%)	9 (25.0)	7 (20.6)	
SMLT, *n* (%)	18 (50.0)	16 (47.1)	
**BCVA (decimal)**			
Baseline	0.52 ± 0.14	0.51 ± 0.15	0.845
Final	0.85 ± 0.12	0.45 ± 0.18	<0.001
Within-group *p*-value	<0.001	0.042	
**Sattler’s layer foci**			
Baseline	57.78 ± 31.79	55.12 ± 21.07	0.674
Final	48.61 ± 24.00	59.74 ± 22.78	0.043
Within-group *p*-value	0.001	0.041	
**Haller’s layer foci**			
Baseline	7.47 ± 10.17	7.91 ± 8.98	0.854
Final	4.28 ± 7.03	7.97 ± 10.96	0.047
Within-group *p*-value	0.039	0.972	
**Central retinal thickness (μm)**			
Baseline	417.86 ± 133.48	408.54 ± 128.76	0.765
Final	245.32 ± 45.67	385.45 ± 112.34	<0.001
Within-group *p*-value	<0.001	0.042	
**Choroidal thickness (μm)**			
Baseline	418.47 ± 98.31	422.35 ± 102.44	0.872
Final	385.24 ± 88.45	428.67 ± 98.76	0.045
Within-group *p*-value	0.028	0.782	
**SHRM resolution,** *n* **(%)**	14/18 (77.8)	6/19 (31.6)	0.002
**pMA development,** *n* **(%)**	4 (11.1)	11 (32.4)	0.001
	**pMA Developed (***n* = **15)**	**No pMA (***n* = **55)**	
**Baseline total HCF count**	89.26 ± 42.15	61.62 ± 35.48	0.089
**Final total HCF count**	94.36 ± 46.82	53.63 ± 26.48	0.003

Data are presented as mean ± standard deviation for continuous variables and as number (percentage) for categorical variables. Within-group p-values represent paired comparisons between baseline and final measurements within each group. Between-group p-values compare responders versus non-responders at each timepoint using independent *t*-tests for continuous variables and chi-square or Fisher’s exact tests for categorical variables.

*PDT* photodynamic therapy, *SMLT* subthreshold micropulse laser therapy, *BCVA* best-corrected visual acuity, *SHRM* subretinal hyperreflective material, *pMA* pachychoroid macular atrophy.

Analysis of response rates among treatment modalities revealed that PDT had achieved a response rate of 45.0% (9/20 patients), eplerenone therapy had demonstrated a response rate of 56.3% (9/16 patients), and SMLT had shown a response rate of 52.9% (18/34 patients). No statistically significant difference in response rates was observed among the three treatment modalities (*p* = 0.742).

### Hyperreflective choroidal foci analysis

Quantification of HCF revealed significant differences between treatment response groups. At baseline, responders demonstrated a mean of 57.78 ± 31.79 foci in Sattler’s layer and 7.47 ± 10.17 foci in Haller’s layer, while non-responders showed 55.12 ± 21.07 and 7.91 ± 8.98 foci, respectively, with no significant differences between groups (*p* = 0.674 and *p* = 0.85,4 respectively) (Table 2).

At the 12-month follow-up, marked differences emerged between groups. Responders showed a significant reduction to 48.61 ± 24.00 foci in Sattler’s layer (*p* = 0.001 compared to baseline) and 4.28 ± 7.03 foci in Haller’s layer (*p* = 0.039 compared to baseline). Conversely, non-responders demonstrated an increase to 59.74 ± 22.78 foci in Sattler’s layer (*p* = 0.041 compared to baseline) while Haller’s layer foci remained stable at 7.97 ± 10.96 (*p* = 0.972 compared to baseline). Between-group comparisons at follow-up revealed significantly different foci counts in both Sattler’s layer (*p* = 0.043) and Haller’s layer (*p* = 0.047).

The intraclass correlation coefficient (ICC) for intergrader agreement was 0.89 (95% CI: 0.84–0.93) for Sattler’s layer foci and 0.87 (95% CI: 0.81–0.91) for Haller’s layer foci, indicating excellent agreement. The ICC for intragrader repeatability (performed by PV) was 0.92 (95% CI: 0.88–0.95) for Sattler’s layer and 0.90 (95% CI: 0.85–0.93) for Haller’s layer.

### Morphological and functional outcomes

Comprehensive analysis of morphological parameters demonstrated distinct patterns between responder and non-responder groups. BCVA improved significantly in responders from 0.52 ± 0.14 to 0.85 ± 0.12 decimal (*p* < 0.001), while non-responders showed deterioration from 0.51 ± 0.15 to 0.45 ± 0.18 decimal (*p* = 0.042). Between-group comparison at follow-up revealed a highly significant difference in final BCVA (*p* < 0.001).

CRT decreased markedly in responders from 417.86 ± 133.48 μm to 245.32 ± 45.67 μm (*p* < 0.001), while non-responders showed a more modest reduction from 408.54 ± 128.76 μm to 385.45 ± 112.34 μm (*p* = 0.042). SFCT demonstrated a significant reduction in responders from 418.47 ± 98.31 μm to 385.24 ± 88.45 μm (*p* = 0.028), whereas non-responders showed no significant change from 422.35 ± 102.44 μm to 428.67 ± 98.76 μm (*p* = 0.782). As expected by definition, SRF height resolved completely in responders (from 304.45 ± 156.78 μm to 0 ± 0 μm, *p* < 0.001), while non-responders showed partial reduction from 298.67 ± 148.92 μm to 145.34 ± 98.76 μm (*p* < 0.001).

### Qualitative outcomes

Analysis of qualitative parameters revealed significant differences in resolution patterns between groups. SHRM, present in 18 responders (50.0%) and 19 non-responders (55.9%) at baseline (*p* = 0.674), showed marked differences in resolution. At follow-up, SHRM persisted in only 4 responders (11.1%) compared to 13 non-responders (38.2%) (*p* = 0.002). Within-group analysis demonstrated significant SHRM resolution in responders (*p* = 0.001) and non-responders (p = 0.042).

pMA development represented a critical outcome measure, with no patients demonstrating pMA at baseline. At 12-month follow-up, pMA had developed in 15 patients (21.4% of the total cohort). Importantly, pMA development showed a significant association with treatment response: 4 responders (11.1%) developed atrophy compared to 11 non-responders (32.4%) (*p* = 0.001). Within-group analysis confirmed significant pMA development in both responders (*p* = 0.045) and non-responders (*p* < 0.001).

Patients who developed pMA showed a trend toward higher baseline foci counts compared to those who did not develop pMA, although these differences did not reach statistical significance. Mean baseline Sattler’s layer foci were 74.38 ± 35.42 in patients who developed pMA versus 54.88 ± 31.79 in those who did not (*p* = 0.162). Similarly, Haller’s layer foci were 14.88 ± 12.17 versus 6.74 ± 8.98, respectively (*p* = 0.063).

### Predictive factors for atrophy development

Linear regression analysis identified several significant predictors of pMA development. Baseline total foci count emerged as the strongest predictor (β = 0.465, *R*² = 0.324, *p* < 0.001), followed by baseline Sattler’s layer foci (β = 0.428, *R*² = 0.283, *p* = 0.001) and baseline Haller’s layer foci (β = 0.312, *R*² = 0.186, *p* = 0.007). Final foci counts demonstrated even stronger associations: final total foci count (β = 0.512, *R*² = 0.348, *p* < 0.001), final Sattler’s layer foci (β = 0.486, *R*² = 0.312, *p* < 0.001), and final Haller’s layer foci (β = 0.365, *R*² = 0.228, *p* = 0.003). Changes in foci were also predicted by atrophy development: Sattler’s layer foci changes (β = 0.415, *R*² = 0.246, *p* = 0.030) and Haller’s layer foci changes (β = 0.386, *R*² = 0.198, *p* = 0.039) (Supplementary Table 1).

Notably, other baseline morphological parameters, including SFCT, CRT, baseline SRF height, presence of SHRM, and disease duration, did not demonstrate significant predictive value for pMA development.

## Discussion

This retrospective study represents the first comprehensive investigation of HCF as layer-specific biomarkers in CSC, providing novel insights into their role as predictors of treatment response and pMA development. The most significant finding of our study is the demonstration that HCF serve as powerful predictors of pMA development, with baseline total foci count explaining 32.4% of the variance in atrophy occurrence.

This relationship represents a paradigm shift in our understanding of CSC pathophysiology, suggesting that choroidal inflammatory or degenerative processes, as reflected by these foci [29, 30], may be fundamental drivers of long-term retinal damage rather than mere epiphenomena. The stronger predictive value of final foci counts compared to baseline values indicates that the temporal evolution of these structures carries even greater prognostic significance, potentially reflecting ongoing disease activity that culminates in irreversible retinal changes.

The differential behavior of hyperreflective foci between treatment responders and non-responders provides compelling evidence for their role as dynamic biomarkers of disease activity. Responders demonstrated a significant reduction in both Sattler’s and Haller’s layer foci, while non-responders showed either stability or an increase in foci counts. This divergent pattern suggests that successful treatment response is associated with resolution of the underlying choroidal pathological processes that generate these hyperreflective structures.

The observation that Sattler’s layer foci showed more pronounced changes than Haller’s layer foci aligns with current understanding of pachychoroid disease pathophysiology [31, 32], where medium-sized choroidal vessels are considered primary targets of the disease process.

Our findings contribute significantly to the growing body of literature on pMA, a recently recognized complication of chronic CSC that shares similarities with geographic atrophy in age-related macular degeneration but demonstrates distinct characteristics [11].

From a pathophysiological perspective, HCF likely represent multiple overlapping processes that reflect chronic disease activity [9, 10, 33]. These structures may correspond to inflammatory infiltrates, lipofuscin deposits, melanophage accumulation, or areas of choroidal vascular remodeling in response to chronic dysfunction [33]. Recent histopathological studies in AMD have demonstrated that similar hyperreflective structures correspond to activated microglia and inflammatory cells, suggesting that choroidal inflammation plays a crucial role in disease progression [34–36].

Our findings align with previous observations by Ruiz-Medrano et al. [33], who demonstrated that hyperreflective choroidal spots were significantly more prevalent in chronic CSC compared to acute forms (83.3% vs 33.3%). These authors suggested that such alterations may reflect a fibrotic response to chronic vascular hyperpermeability and inflammation in the choroid, supporting our hypothesis that HCF represent biomarkers of ongoing pathological processes. In the context of CSC, these foci may represent the choroidal component of a systemic inflammatory response that manifests clinically as chronic serous detachment and ultimately progresses to irreversible retinal atrophy.

The markedly higher rate of atrophy development in treatment non-responders compared to responders underscores the critical importance of achieving complete fluid resolution in chronic CSC. This finding challenges the traditional view that partial response to treatment represents an acceptable clinical outcome, suggesting instead that persistent subretinal fluid may serve as a marker of ongoing disease activity that predisposes to long-term complications. The observation that even patients with complete fluid resolution can develop atrophy indicates that damage may occur despite apparent treatment success, highlighting the need for continued monitoring and potentially more aggressive therapeutic interventions in high-risk patients identified through baseline foci quantification.

An important distinction must be made regarding the predictive value of HCF in our study. While baseline HCF counts were significant predictors of pMA development, they did not predict treatment response, as demonstrated by the absence of baseline differences between responders and non-responders (*p* = 0.674 and *p* = 0.854 for Sattler’s and Haller’s layers, respectively). Rather, the temporal evolution of HCF during follow-up correlated with treatment outcomes, with responders showing significant foci reduction and non-responders demonstrating stability or increase. This finding suggests that baseline HCF quantification serves primarily as a prognostic tool for identifying patients at risk of irreversible atrophic changes, while HCF changes over time may reflect treatment efficacy and ongoing disease activity.

Our results have important implications for treatment selection and monitoring strategies in chronic CSC. The ability to quantify hyperreflective foci provides clinicians with an objective tool for assessing disease severity and predicting long-term outcomes at the time of initial presentation. Patients with high baseline foci counts may benefit from more aggressive treatment approaches or closer monitoring for early signs of pMA development. Furthermore, the temporal changes in foci counts during treatment could serve as early biomarkers of treatment efficacy, potentially allowing for treatment modification before irreversible changes occur.

The incorporation of Chhablani’s multimodal imaging-based classification system into our methodology represents an important step toward standardized CSC nomenclature and outcome assessment [3]. From a broader perspective, our findings contribute to the growing understanding of choroidal biomarkers in retinal disease. The success of HCF quantification in predicting CSC outcomes parallels similar observations in AMD, DR, and inherited retinal diseases. This convergence suggests that choroidal inflammatory and degenerative processes may represent common pathways in multiple retinal conditions, opening new avenues for cross-disease research and therapeutic development. Additionally, the potential role of anti-inflammatory therapies in reducing foci burden and preventing atrophy development represents an attractive area for therapeutic intervention studies.

The integration of artificial intelligence and machine learning approaches into foci quantification represents another promising research direction. Automated detection and quantification algorithms could eliminate inter-observer variability and enable large-scale population studies that would be impractical with manual counting methods.

Several limitations of our study must be acknowledged when interpreting these results. The retrospective design introduces potential selection bias and limits our ability to control for confounding variables that may influence treatment outcomes. The relatively short follow-up period of 12 months may not capture the full spectrum of long-term complications, particularly given that pMA development can occur years after initial presentation. The lack of detailed topographic correlation between foci location and specific areas of pMA development represents a missed opportunity to better understand the spatial relationship between these biomarkers and clinical outcomes. Additionally, the manual quantification of hyperreflective foci, while performed by experienced graders with good inter-observer agreement, introduces inherent subjectivity that may limit reproducibility across different centers and is significantly time-consuming, potentially limiting its widespread clinical adoption. Finally, the single-center design and predominantly Caucasian population may limit the generalizability of our findings to other demographic groups and healthcare settings.

Despite these limitations, our study provides compelling evidence for the clinical utility of hyperreflective choroidal foci quantification in chronic CSC management. The demonstration that these morphological biomarkers predict both treatment response and atrophy development establishes a foundation for their integration into clinical practice and future research protocols.

### What was known before:

- Central serous chorioretinopathy (CSC) is associated with choroidal vascular abnormalities and can lead to irreversible visual loss through the development of retinal atrophy development.
- Hyperreflective choroidal foci have been observed in various chorioretinal conditions and are associated with inflammatory processes.
- Chronic CSC can develop pachychoroid macular atrophy (pMA), but predictive factors for atrophy development remain poorly understood.
- Previous studies showed hyperreflective spots were more prevalent in chronic versus acute CSC (83.3% vs 33.3%), but quantitative layer-specific analysis was lacking.
- Treatment response prediction and identification of high-risk patients for atrophy development remained challenging in chronic CSC management.

### What this study adds:

- First comprehensive layer-specific quantification of hyperreflective choroidal foci in Sattler’s and Haller’s layers as prognostic biomarkers in chronic CSC.
- Baseline total foci count is the strongest predictor of pachychoroid macular atrophy development (β = 0.465, *R*² = 0.324, *p* < 0.001).
- Hyperreflective foci demonstrate dynamic behavior: responders show significant foci reduction while non-responders show increased foci counts.
- Patients with complete treatment response can still develop atrophy (11.1%), highlighting the need for continued monitoring even in apparent treatment success.
- Temporal evolution of foci carries greater prognostic significance than baseline counts alone, with final foci counts showing stronger associations with atrophy (*R*² = 0.348).
- Layer-specific analysis reveals Sattler’s layer foci show more pronounced changes than Haller’s layer foci, aligning with pachychoroid disease pathophysiology.

## Supplementary information

Supplementary Table 1

## Data Availability

The datasets generated and analyzed during the current study are not publicly available due to privacy concerns and regulations regarding patient data protection, but de-identified data are available from the corresponding author upon reasonable request and with appropriate institutional review board approval.
