# Supplementary material for: Hyperreflective choroidal foci may predict pachychoroid macular atrophy development in central serous chorioretinopathy
Source: Eye (Lond). 2026 Feb 5;40(5):689–96. doi: 10.1038/s41433-026-04277-8 (PMC13013961; doi:10.1038/s41433-026-04277-8)
Supplement: Supplementary file 1 — Supplementary Table 1 [file 41433_2026_4277_MOESM1_ESM.docx]

| **Supplemental Table 1. Predictive Factors for Pachychoroid Macular Atrophy Development** | | | |
| --- | --- | --- | --- |
|  | **β Coefficient** | **R²** | **P-value** |
| **Baseline Hyperreflective Foci** |  |  |  |
| Sattler's layer foci | 0.428 | 0.283 | 0.001 |
| Haller's layer foci | 0.312 | 0.186 | 0.007 |
| Total foci count | 0.465 | 0.324 | <0.001 |
| **Final Hyperreflective Foci** |  |  |  |
| Final Sattler's layer foci | 0.486 | 0.312 | <0.001 |
| Final Haller's layer foci | 0.365 | 0.228 | 0.003 |
| Final total foci count | 0.512 | 0.348 | <0.001 |
| **Foci Changes (Δ)** |  |  |  |
| Δ Sattler's layer foci | 0.415 | 0.246 | 0.030 |
| Δ Haller's layer foci | 0.386 | 0.198 | 0.039 |
| **Other Baseline Parameters** |  |  |  |
| Choroidal thickness | 0.156 | 0.089 | 0.245 |
| Central retinal thickness | 0.142 | 0.076 | 0.312 |
| Subretinal fluid height | 0.134 | 0.068 | 0.438 |
| SHRM presence | 0.224 | 0.115 | 0.224 |
| Disease duration | 0.245 | 0.124 | 0.178 |
| Analysis performed on patients without baseline atrophy (n=70). **β coefficient** = standardised regression coefficient indicating the strength and direction of association; **R² =** coefficient of determination representing the proportion of variance in atrophy development explained by each variable; **Δ =** change from baseline to 12-month follow-up (final value minus baseline value). All regression models were adjusted for age and sex. ***SHRM =*** subretinal hyperreflective material. | | | |
